# SARS-CoV-2 seroprevalence in the city of Puerto Madryn: Underdiagnosis and relevance of children in the pandemic

**DOI:** 10.1371/journal.pone.0263679

**Published:** 2022-03-14

**Authors:** Daniel Schonfeld, Hugo Fernández, Julio Ramírez, Denisse Acosta, Julián Becerra, Magali Wettstein, Teresa Strella, Marcelo Vaccaro, Sergio Arias, Vilma Rodríguez Calvo, Roberto Neme, Daniel Pérez-Chada

**Affiliations:** 1 Centro Diagnostico San Jorge, Puerto Madryn, Chubut, Argentina; 2 Instituto Nacional de Enfermedades Respiratorias “Dr. Emilio Coni”, Administración Nacional de Laboratorios e Institutos de Salud “Dr. Carlos G. Malbran”, Santa Fe, Argentina; 3 School of Medicine Louisville, Louisville, Kentucky, United States of America; 4 Hospital de Puerto Madryn, Puerto Madryn, Chubut, Argentina; 5 Subsecretaria de Atención Primaria, Puerto Madryn, Chubut, Argentina; 6 Epidemiologia del Ministerio de Salud, Rawson, Chubut, Argentina; 7 Universidad Austral, Hospital Universitario Austral, Pulmonary Medicine, Pilar, Argentina; Shahrood University of Technology, ISLAMIC REPUBLIC OF IRAN

## Abstract

**Background:**

Reported cases of COVID-19 may be underestimated due to mild or asymptomatic cases and a low testing rate in the general population.

**Research question:**

What is the seroprevalence of SARS-CoV-2 infection in the general population and how it compares with the data on SARS-CoV-2 cases reported by a national health surveillance system (SNVS 2.0).

**Study design and methods:**

This was a population-based, seroepidemiological, cross-sectional study in the city of Puerto Madryn, a middle size city in the Province of Chubut, Argentina. The study period was between March 3 and April 17, 2021. The sample size was calculated using the technique of calculation of confidence intervals for a proportion. Participants were selected using stratified and cluster probability sampling. A total of 1405 subjects were invited to participate in the study. Participants were divided into the following four age groups: 1) 0 to 14, 2) 15 to 39, 3) 40 to 64, and 4) 65 or older. After informed consent was obtained, a blood sample was taken by puncture of the fingertip, and a structured questionnaire was administered to evaluate demographics, socioeconomic status, level of education, comorbidities and symptoms suggestive of COVID-19.

COVID-19 seroprevalence was documented using an immunoenzymatic test for the in vitro detection of IgG antibodies specific to the spike protein of SARS-CoV-2.

**Results:**

A total of 987 participants completed the survey. Seropositivity in the full study population was 39,2% and in those under 15 years of age, 47.1%. Cases reported by the SNSV 2.0 amounted to 9.35% of the total population and 1.4% of those under 15 years of age.

**Interpretation:**

The prevalence of COVID-19 infection in the general population is four times higher than the number of cases reported by the SNVS 2.0 in the city of Puerto Madryn. For each child under the age of 15 identified by the SNVS 2.0 with COVID-19, there are more than 30 unrecognized infections. Seroepidemiological studies are important to define the real extent of SARS-CoV-2 infection in a particular community. Children may play a significant role in the progression of the current pandemic.

## Introduction

Coronaviruses are important human and animal pathogens; there are seven known types that can infect humans. Four of them cause different varieties of the common cold, while another three, which have recently appeared, produce much more serious disorders, such as the Severe Acute Respiratory Syndrome (SARS), which appeared in 2002; the Middle East Respiratory Syndrome (MERS), which emerged in 2012 and finally this new disease, COVID-19, caused by SARS-CoV-2 [1].

Inadequate knowledge about the scope of the 2019 coronavirus pandemic (COVID-19) interferes with the health system planning and response. The seroprevalence of the disease in a region or city is critical to adjust the health care system response. These studies are the most effective means to detect the number of asymptomatic and oligosymptomatic cases, enabling the health providers to identify a group of individuals who are often undiagnosed, but who nevertheless play a key role in the spread and maintenance of the pandemic [2, 3].

Based on the seroprevalence patterns, it is possible to understand whether viral shedding revalidates or refutes preconceptions in this regard [4–6], being an extremely important factor for carrying out mitigation measures, as well as for planning the distribution of vaccines [7]. Likewise, and if their numbers were large enough, they could be an epidemiological barrier to the spread of the disease (herd immunity) [8].

In Argentina, most reports of confirmed cases arise from tests based on polymerase chain reaction (PCR), antigen tests, and epidemiological clinical criteria [9, 10].

In the city of Puerto Madryn, the first positive case was diagnosed on June,10, 2020. The increase in the number of cases was observed from epidemiological week 34, with 11,171 cases diagnosed as of April,17,2021. The aim of the present study (SeroMadryn study) is to explore the prevalence of Sars-Cov-2 infection in the city of Puerto Madryn through the measurement of specific IgG antibodies and characterizing the affected population and to compare the data with SARS-CoV-2 cases reported by a national health surveillance system (SNVS 2.0).

## Methods

Our protocol was based on a World Health Organization protocol for COVID-19 antibody testing at the population level [11]. Organizers of the study were the Ministry of Health of the Province of Chubut, Undersecretary of Health of the city of Puerto Madryn and the National Institute of Respiratory Diseases "Emilio Coni".

This is a population-based, sero-epidemiological, cross-sectional study to assess the prevalence of antibodies to SARS-CoV-2. The target population of the study were subjects of different age groups residing in the city of Puerto Madryn. Were excluded from the study subjects who refused to give their informed written consent, those in whom digital blood puncture was contraindicated, subjects with acute symptoms potentially related to COVID-19 infection, subjects who received COVID-19 vaccination, and subjects with mental disorders or unable to make decisions that are not accompanied by their legal representative.

The study period was between March 3 and April 17, 2021. Participants were enrolled from all neighborhoods of the city. Neighborhood boundaries were defined according to the primary health care centers (CAPS) of the city of Puerto Madryn.

The sample size was calculated using the technique of "calculation of confidence intervals for a proportion". For the calculation of the sample size, the estimated population of inhabitants in the city of Puerto Madryn in 2020 were 115,353 [12]. Since the information on seroprevalence was unknown, we used the prevalence estimated by seroprevalence studies of COVID-19 performed in the city of Buenos Aires (10.1%) [13] and in the Buenos Aires province (14.8%) [14]. Considering this proportion, with a confidence level of 95%, a precision of 2.4%, and a design effect of 1.5, a sample size of 1,088 subjects was invited to participate.

Participants were selected using stratified and cluster probability sampling. The stratification criterion was established according to the size of the geographic units of the city of Puerto Madryn, which were represented by the population enrolled in each primary health care center. The distribution of the population by age group, (0 to 14, 15 to 39, 40 to 64, 65 and older) was calculated based on province of Chubut age distribution and according to the recommendations in the WHO protocol [11].

An awareness campaign and advertisement in the local media (radio, TV, etc.) was implemented to inform the citizens about the nature of the study. A structured questionnaire was used to evaluate demographics, socioeconomic status, level of education, comorbidities and symptoms suggestive of COVID19. Study personnel was in charge of the field procedures, which included reception of the list of the random selection of the population, distributing and collecting study questionnaires, informed written consents, and an operational guide for the correct collection, storage, as well as transporting the blood samples for processing and analysis at the central laboratory of the Hospital Zonal Dr. Andrés Ísola.

All procedures performed in this study were in accordance with the ethical standards of the national research committee and with the 1964 Helsinki declaration and its later amendments or comparable ethical standards. The protocol was approved by the Comité de Bioética del Area Programática Norte, approval note number 001/2021.

Informed written consent was obtained from all individual participants included in the study. In the case of the minors the informed written consent was obtained from their parents or guardians.

Processing and analysis were conducted according to current regulations. The COVIDAR IgG® test consists of an immunoenzymatic test using a blood sample taken by puncture of the fingertip; the test is heterogeneous, non-competitive, and based on an indirect method for the in vitro detection of IgG antibodies specific to the spike protein of SARS-CoV-2 in human serum or plasma samples. The test has a specificity of 100% in pre-pandemic samples, 74% in patient samples RT-PCR positive for SARS-CoV-2 within the first three weeks after the onset of symptoms, and at least 91% in samples more than 21 days from the onset of symptoms [15].

The covariates used were demographic and self-reported health history, symptoms related to COVID-19, characteristics of living situation, the number of cohabitants and previous diagnosis of COVID-19.

Seroprevalence was calculated as the proportion of samples that were confirmed as reactive, stratified by sex and age group (0 to 14, 15 to 39, 40 to 64, 65 and older). 95% confidence intervals (95% CI) were estimated.

Statistical analyses were performed using the R version 3.6.1 (R Foundation for Statistical Computing). Results were reported as absolute values and percentages, and p values were evaluated using Fisher’s exact test. Two-sided p values less than 0.05 were considered statistically significant.

## Results

**Fig 1:**
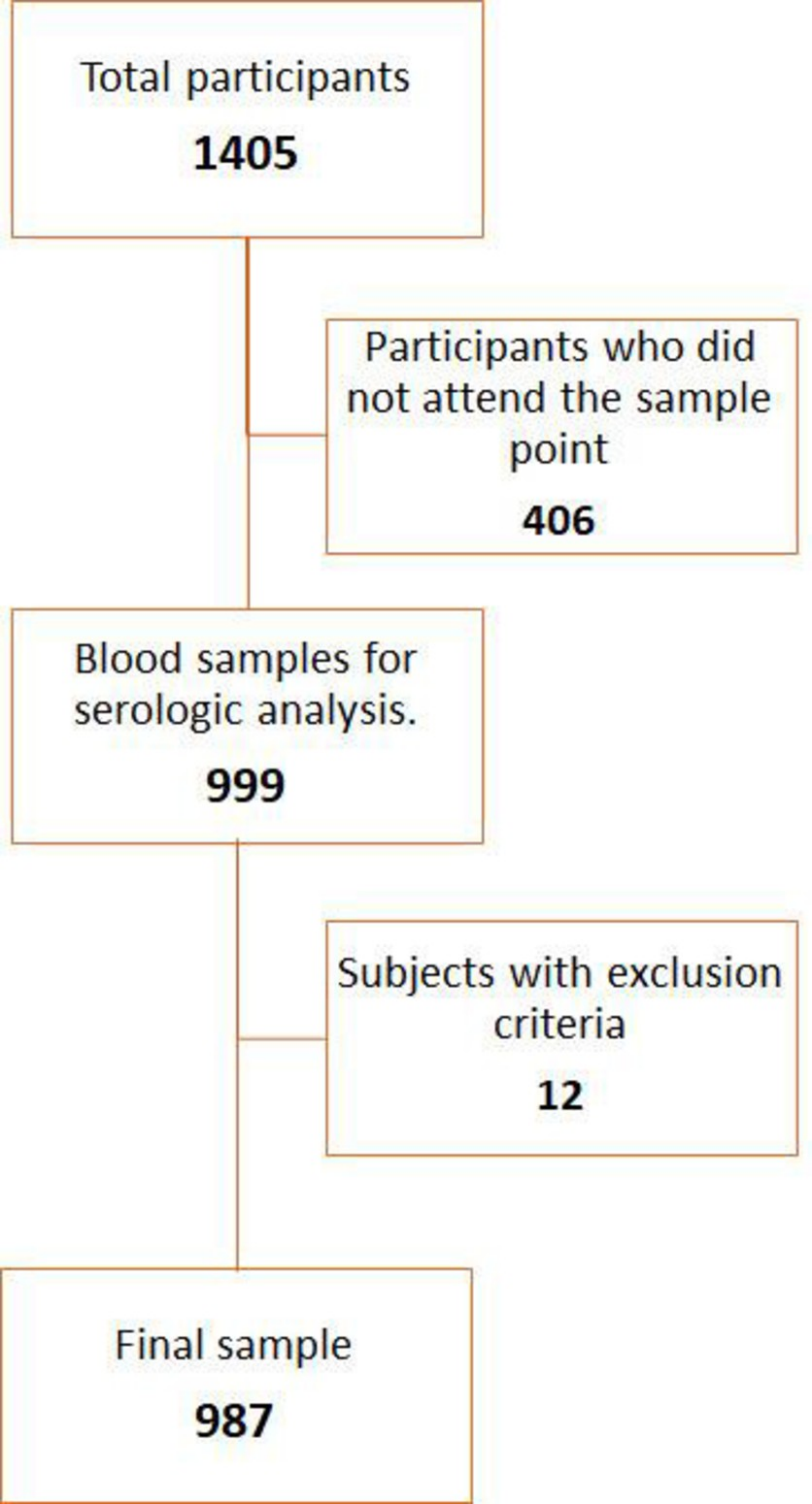
Flowchart of the participants who completed the survey and attended the sampling point to perform the serological test.

During the recruitment period, 1,405 subjects gave their informed consent to participate in the study and responded to the survey, a final sample of 987 citizens were included in the final analysis. Adherence to the study was 70.3% (Fig 1).

Table 1 shows the characteristics of the studied population and the comparison with the population distribution of the city of Puerto Madryn.

**Table 1:** Sociodemographic and clinical characteristics of the studied sample and relationship with the population estimates of Puerto Madryn.

		Sample		Population
		Number	Percentage	Percentage
**Total**		**987**		
**Sex**	Men	360	36,5%	51,9%
	Women	627	63,5%	48,9%
**Age group**	Under 15	227	23,0%	25,0%
	15–39	342	34,7%	38,7%
	40–64	337	34,1%	27,2%
	65 and older	81	8,2%	9,2%
**Geographical area**	CAPS 1	83	8,4%	8,4%
	CAPS 2	127	12,9%	15,3%
	CAPS 3	69	7,0%	8,4%
	CAPS 4	164	16,6%	16,8%
	CAPS 5	85	8,6%	8,4%
	CAPS 6	99	10,0%	9,2%
	CAPS 7	112	11,3%	7,6%
	CAPS 8	153	15,5%	16,8%
	CAPS 9	95	9,6%	9,2%
Level of education*	None	3	0,4%	
	Incomplete primary	38	5,2%	
	Full primary	139	19,2%	
	Incomplete secondary	140	19,3%	
	Full secondary	189	26,1%	
	Incomplete tertiary	37	5,1%	
	Full tertiary	75	10,4%	
	Incomplete university	57	7,9%	
	Full university	39	5,4%	
	N/A	7	1,0%	
Occupation*	Housewife	165	22,8%	
	Salaried worker	224	30,9%	
	Retired/Pensioner	101	14,0%	
	Student	66	9,1%	
	Unemployed	57	7,9%	
	Non-salaried worker	37	5,1%	
	Other	60	8,3%	
	N/A	14	1,9%	
**Cohabitants in the home**	0	17	1,7%	
	1	109	11,0%	
	2	187	18,9%	
	3	205	20,8%	
	4	227	23,0%	
	5 or more	232	23,5%	
	N/A	10	1,0%	
**Number of rooms in the house**	1	134	13,6%	
	2	395	40,0%	
	3	316	32,0%	
	4 or more	128	13,0%	
	N/A	14	1,4%	
**Previous diagnosis of Covid-19**	Yes	197	20,0%	
	No	775	78,5%	
	N/A	15	1,5%	
**Comorbidities**	Yes	384	38,9%	
	No	603	61,1%	
**Presence of symptoms**	Yes	584	59,2%	
	No	403	40,8%	

*Over 18 years old.

The sample consisted of 63.5% women. The mean (standard deviation) age of the entire cohort was 34.5 years (± 19.7). The distribution by age groups in the studied sample compared to the population was 6.9% higher in the group aged 40 to 64 years. Cohabitation with 3 or more persons was detected in 67.3% of the sample. Almost 1 in 4 people included in the study had 5 or more cohabitants (23.5%), While 40.0% of the cases studied lived in a house with two rooms, and 13.6% reported a single room in the home. Combining these variables, we found that 4.3% of the people surveyed lived in critical overcrowding condition (more than 3 people per room), and almost 70% were not overcrowded (2 people per room).

Comorbidities or risk factors were reported by 38.9% of the studied population; being hypertension (13.7%) and smoking (12.5%) the most common. One or more symptoms related to COVID-19 infection was reported by 59.2% of the people surveyed during the study period. Fatigue was the most common symptom (53.2%), followed by nasal congestion (53.1%) and sore throat (49.6%).

History of previous COVID-19 infection was reported by 20% of the responders. Diagnoses was confirmed by nasal swab in 39.6% of cases, 34.0% by epidemiological link, and 16.8% by symptoms of anosmia or dysgeusia (Table 1).

The prevalence of IgG antibodies against Sars-Cov2 found in this study was 39.2% (95%CI:32.6,42.3).

Table 2 shows seroprevalence stratified by the sociodemographic and clinical characteristics of the individuals studied.

**Table 2:** COVID-19 seroprevalence in the Puerto Madryn population studied.

Characteristics		Total	Reagents for SARS-CoV-2	Seroprevalence (95% CI)
**Total**		**987**	**387**	**39,2 (36,2–42,3)**
**sex**	Men	360	141	39,2 (34,1–44,2)
	Women	627	246	39,2 (35,4–43,1)
**Age group**	Under 10	114	49	43,0 (33,2–52,1)
	10–14	113	58	51,3 (42,1–60,5)
	15–39	342	119	34,8 (29,7–39,8)
	40–64	337	129	38,3 (33,1–43,5)
	65 and older	81	32	39,5 (28,9–50,2)
**Geographical area**	CAPS 1	83	31	37,3 (26,9–47,8)
	CAPS 2	127	50	39,4 (30,9–47,9)
	CAPS 3	69	25	36,2 (24,9–47,6)
	CAPS 4	164	68	41,5 (33,9–49,0)
	CAPS 5	85	42	49,4 (38,8–60,0)
	CAPS 6	99	34	34,3 (25,0–43,7)
	CAPS 7	112	29	25,9 (17,8–34,0)
	CAPS 8	153	71	46,4 (38,5–54,3)
	CAPS 9	95	37	38,9 (29,1–48,8)
Level of education*	None	3	0	0,0
	Incomplete primary	38	20	52,6 (36,8–68,5)
	Full primary	139	56	40,3 (32,1–48,4)
	Incomplete secondary	140	47	33,6 (25,7–41,4)
	Full secondary	189	70	37,0 (30,2–43,9)
	Incomplete tertiary	37	17	45,9 (29,9–62,0)
	Full tertiary	75	17	22,7 (13,2–32,1)
	Incomplete university	57	21	36,8 (24,3–49,4)
	Full university	39	12	30,8 (16,3–45,3)
	N/A	7	4	
Occupation*	Housewife	165	70	42,4 (34,9–50,0)
	Salaried worker	224	68	30,4 (24,3–36,4)
	Retired/retired	101	32	31,7 (22,6–40,8)
	Student	66	30	45,5 (33,4–57,5)
	unoccupied	57	21	36,8 (24,3–49,4)
	Non-salaried worker	37	17	45,9 (29,9–62,0)
	Other	60	20	33,3 (21,4–45,3)
	N/A	14	6	42,9 (16,9–68,8)
**Cohabitants in the home**	Less than 3	313	106	33,9 (28,6–39,1)
	3 a 4	432	176	40,7 (36,1–45,4)
	5 and more	232	100	43,1 (36,7–49,5)
	N/A	10	5	
**Number of rooms in the house**	1	134	49	36,6 (28,4–44,7)
	2	395	115	29,1 (24,6–33,6)
	3	316	128	40,5 (35,1–45,9)
	4 or more	128	49	38,3 (29,9–46,7)
	N/A	14	6	
**Previous diagnosis of Covid-19**	Yes	197	140	71,1 (64,7–77,4)
	No	775	239	30,8 (27,6–34,1)
	N/A	15	8	
**Health history**	Yes	384	131	34,1 (29,4–38,9)
	No	603	256	42,5 (38,5–46,4)
**Presence of symptoms**	Yes	584	268	45,9 (41,8–49,9)
	No	403	119	29,5 (25,1–34,0)

The seroprevalence between men and women was equal (39.2%). In children under 15 years of age, seroprevalence was 47.1% (95% CI: 40.5, 53.8). A significant difference was observed between the seroprevalence of children under 15 years of age compared to the 15 to 39 years (p = 0.032) and 40 to 64 years (p = 0.006) groups. No significant difference was observed compared to the result of those aged 65 years and over (p = 0.291). Between the groups aged 15 years and over, no significant differences were found in the percentage of seropositive cases (p > 0.05).

**Fig 2:**
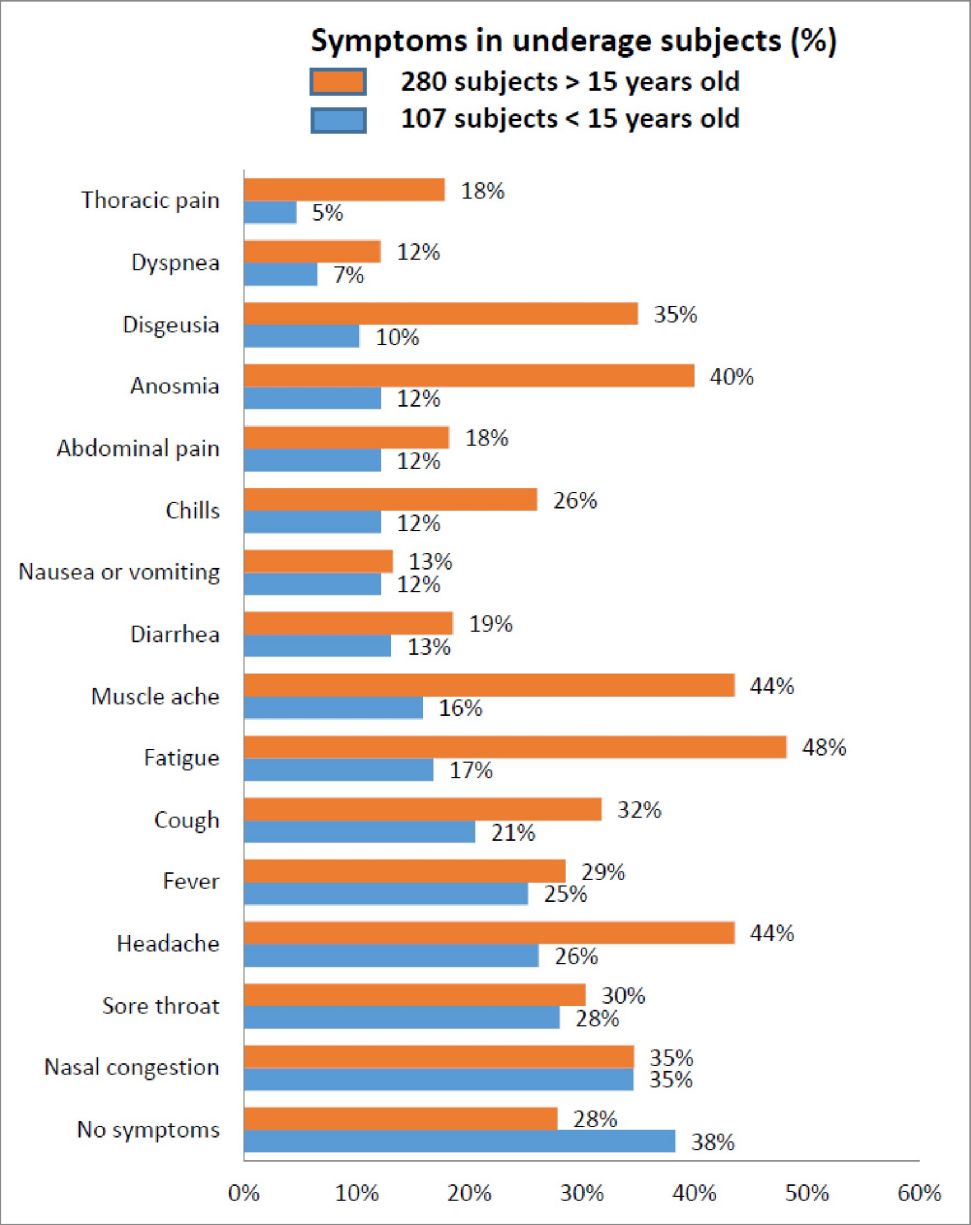
Frequency of symptoms in underaged subjects.

To explore the seroprevalence data from underage participants in more detail we divided the sample into those below 10 years and 10–14 years old. In this study, children under 15 years and particularly those under 10 years old with serpositive test for SARS-CoV-2 reported very few symptoms (Fig 2).

In a group of 227 subjects of which 107 were seropositive for SARS-CoV-2 (49 children under 10 years old and 58 children between 10 and 14 years old). Sore throat, dysgeusia, anosmia and chills were more frequent in the older group showing a statistically significant difference (p<0.05) (S1 Table).

The prevalence of infection by level of education was higher in adults with incomplete primary education: 52.6% (CI: 36.4, 68.7). Adults with incomplete tertiary education and those who completed primary education also had a seroprevalence > 40%. The lowest prevalence was recorded in adults with complete tertiary education 22.7% (CI: 14.1, 34.1). Seroprevalence was significantly higher in people with incomplete primary education than those with complete tertiary education (p = 0.003).

Subjects who reported being students and non-salaried workers were those with the highest prevalence of antibodies, 45.9% (CI: 29.8; 62.9) and 45.5% (CI: 33.3; 58.1), respectively. At the other extreme were salaried workers with a seroprevalence of 30.4% (CI: 24.5, 36.9). The differences between the prevalence in students / non-salaried workers and salaried workers, was not significant (p> 0.05).

Respondents who reported 5 or more cohabitants in the home had a prevalence of infection of 43.1% (CI: 36.7; 49.7), significantly higher than the prevalence of antibodies in individuals living with fewer than 3 people, 33.9% (CI: 28.7, 39.4), p = 0.035.

The highest prevalence of antibodies was observed in people who reported living in dwellings with more than 3 people per room 45.2% (CI: 30.2; 61.2); however, the difference was not significant when compared with the 2 other groups (p = 0.500).

The individuals who reported symptoms related to COVID-19 had a seroprevalence of 45.9% (CI: 41.8, 50.0) compared with 29.5% (CI: 25.0, 34.0) in those who did not report symptoms, this difference being statistically significant (p <0.001) (Table 2).

In subjects who reported comorbidities or risk factors, seroprevalence was 34.1% (CI: 29.4, 31.1) compared to 42.5% (CI: 38.5, 46.5) in those who did not report comorbidities or risk factors, this difference being statistically significant (p = 0.011).

**Fig 3:**
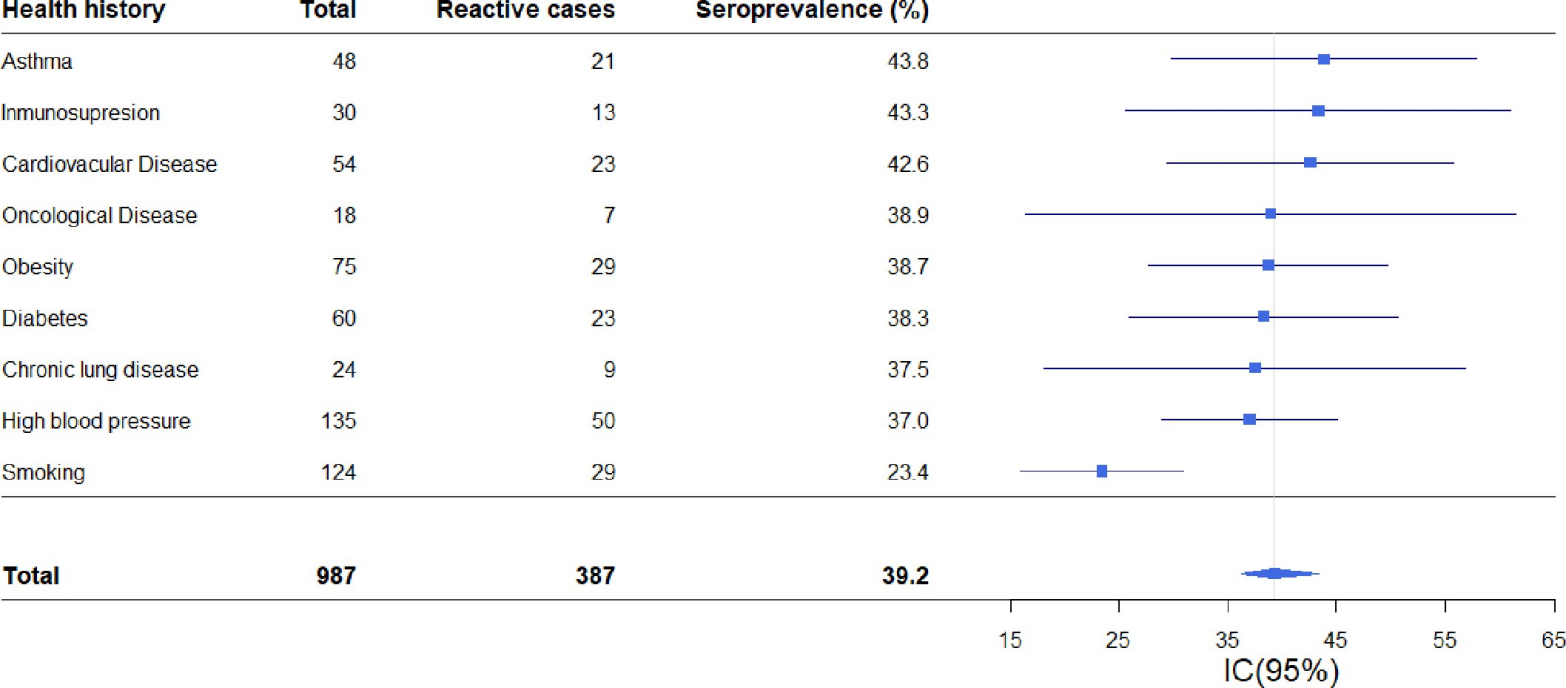
Forest plot of SARS-CoV-2 seroprevalence based on reported health history. Puerto Madryn-Chubut, 2021. N = 987.

People with a history of smoking showed the lowest seroprevalence 23.4% (CI: 16.4, 32.0) with respect to the mean, while seroprevalence among cases with high blood pressure and obesity was 37.0% (CI: 29.0; 45.8) and 38.7% (CI: 27.8, 50.6), respectively. Among the less common comorbidities, the highest seroprevalence was observed in people with asthma (43.8% [CI: 29.8, 58.7]) and cardiovascular disease (41.5% [CI: 29.5, 56.7]) (Fig 3).

**Fig 4:**
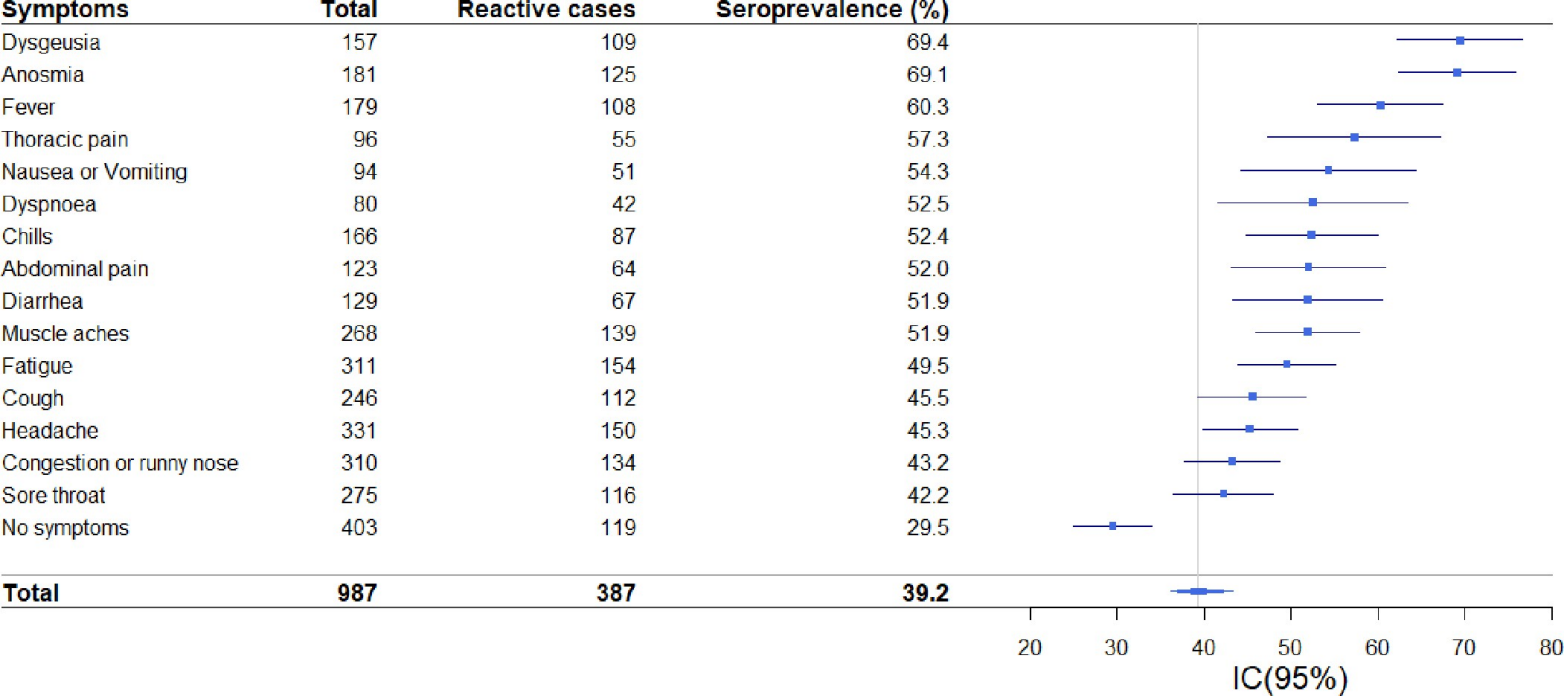
Forest diagram of SARS-CoV-2 seroprevalence according to reported symptoms. Puerto Madryn-Chubut, 2021. N = 987).

Among the symptoms reported in the total sample, anosmia, and dysgeusia were associated with a higher prevalence of reactive cases. SARS-CoV-2 antibodies were found in 69.1% (CI: 61.7, 75.5) of the cases with anosmia and 69.4% (CI: 61.5, 76.4) of the cases with dysgeusia. These symptoms were followed by fever 60.3% (CI: 52.5, 67.5). (Fig 4)

Of the reactive cases, 30.7% reported being asymptomatic during the study period. Fatigue, muscle pain, nasal congestion, and anosmia were the most frequent in more than 30% of the cases (Fig 4).

Of the cases that reported symptoms, 13.7% (80/584) sought health care, seroprevalence in this group being 57.5% (CI: 45.9, 68.3). People who reported not seeking health care had a seroprevalence of 44.1% (CI: 39.3, 48.9), the differences between groups being statistically significant (p = 0.037).

**Fig 5:**
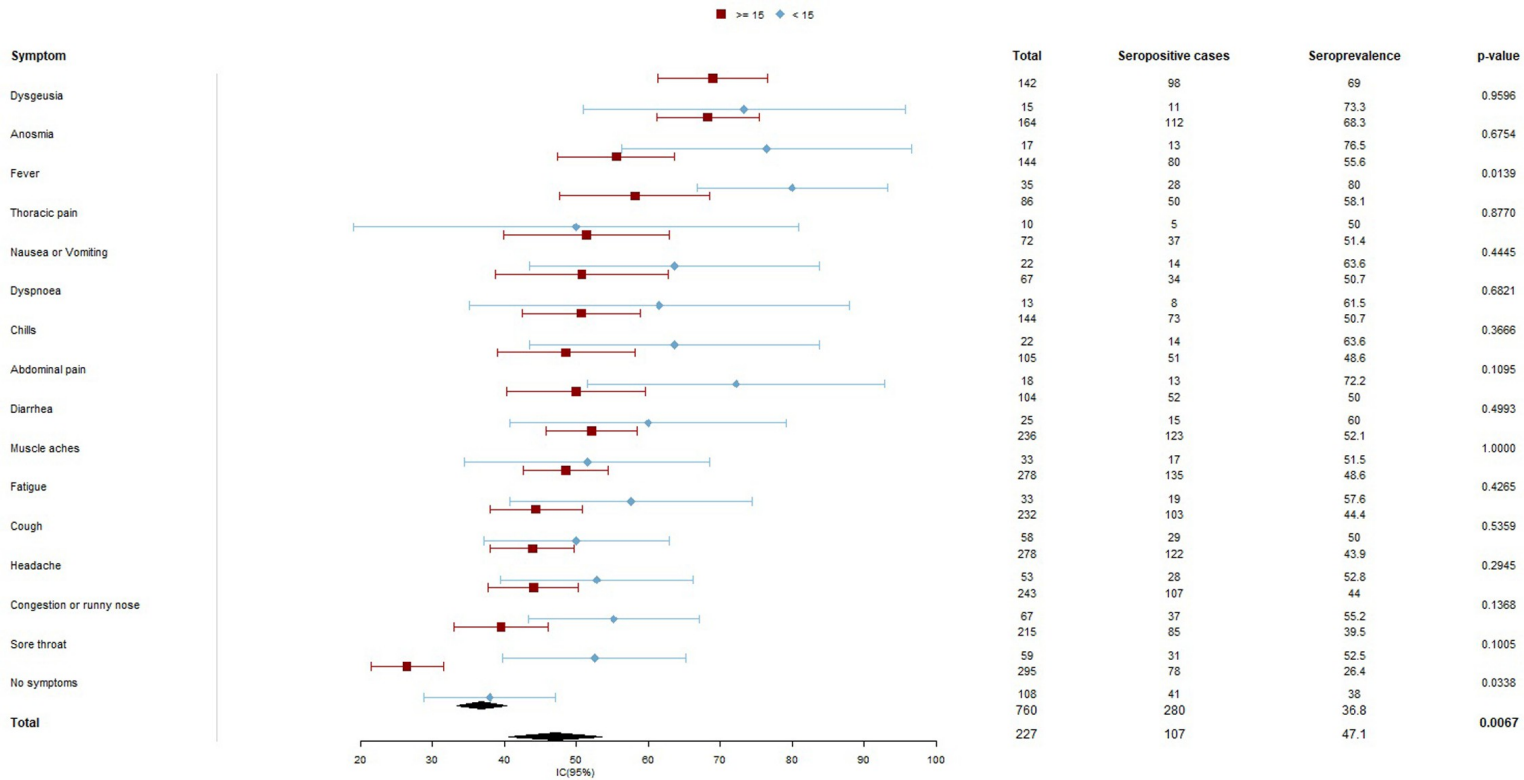
Forest diagram of SARS-CoV-2 seroprevalence according to reported symptoms in two age groups (under 15 and 15 and older). Puerto Madryn-Chubut, 2021. N = 987.

Differences in symptoms between minors under 15 years old and older than15 years with positive result to SARS-CoV-2, are shown in Fig 5.

Comparison between the seroprevalence of SARS-CoV-2 for each symptom in the two age groups shows that there are only statistically significant differences in seroprevalence for fever and no symptoms.

## Discussion

The present study reveals a seroprevalence rate of 39.2% in the general population. As of the closing date of our study, only 9.3% of the population had a confirmed diagnosis of COVID-19 in the national health surveillance system (SNVS 2.0), indicating that for each case diagnosed, there were at least 3 undiagnosed. In those under 15 years of age, positivity was 47.1% of cases, of which only 1.4% were reported in the SNVS 2.0, which indicates that for each diagnosed case there were more than 33 undiagnosed. These findings demonstrate the highly significant rate of underdiagnosis of SARS-CoV-2 infection and indicate that the role played by minors in transmitting the infection could also be important.

The seropositivity found in our study was higher than those reported by other studies conducted in different cities. This difference could be due to the methodology we used in the selection of participants and/or the evolution of the pandemic at different times during the study [16–19].

Figar et al. showed higher prevalence of seropositivity, but with the caveat that this study was carried out on the basis of selected samples in a low-income neighborhood with a high population density [20].

Anosmia and dysgeusia were the most frequent symptoms among reactive cases, although at somewhat lower rates than that reported by Lechien [21]. When we analyze gastrointestinal symptoms among patients with positive serology, it represents 15% of the seropositive subjects, similar figures were reported in different studies [22, 23].

In the present study more than 30% of the seropositive cases were asymptomatic. In the narrative description from Oran et al. summarizing several reports of positive seroprevalence for SARS-CoV-2 in asymptomatic patients, serpositivity ranged from 6% to 96%. The prevalence asymptomatic seropositive patients in our study was greater than the 20% reported by Buitrago-Garcia in a meta-analysis. However, after adjusting by population density the seroprevalence was similar to our study [2, 24].

More than 60% of the positive individuals did not present a pathological history, probably because of the high percentage of young persons in our city who represented in this study. Among those who reported previous pathologies, cardiovascular disease, hypertension and diabetes were the most frequent. In this cohort those with a history of smoking showed the lowest seroprevalence, a finding also reported in an Argentine case study [25] and in other epidemiological studies [26–29].

An important finding of our study is that the highest seropositivity was found in the group of children under 15 years of age (47.1%), of which only 1.4% were reported by the National Health Surveillance System. Our results are substantially higher than those reported in other studies of epidemiological conditions in this population [30–36]. In this age group, we found that four out of five positive cases had no previous diagnosis, even though more than 60% had had at least one symptom of the disease. This finding indicates that we must increase our clinical skills to detect the infection in this age group in order to decrease the potential risk of transmission.

We previously reported 6.6% seropositivity in the population of Argentina diagnosed with COVID-19 by PCR in the group aged 0 to 18 years [25].

The seroprevalence found in this age group is consistent with those reported in other respiratory viruses, such as seasonal flu and H1N1 influenza, for which the highest number of cases are reported among young people [37].

These results are important to highlight the impact of underdiagnosis that occurs in children and adolescents in our population. The finding about the predominance of infection in this age group may be due to the fact that, being a group with low morbidity and mortality [38–41], the search for seroprevalence is not performed in regular basis, however, it may be a transmission factor unidentifiable at home or at school settings. This will be a point to assess, as proposed by Lipsitch, when we analyze whether the risks posed by school closures for the well-being and education of children are justified [42]. Likewise, it is noteworthy that at the time of writing this document, this age group is not receiving the SARS-CoV-2 vaccine, according to the national vaccination plan in force to date [43].

As an additional risk factor for acquire the infection, we found that not having completed primary education, being an informal worker and sharing a home with more than five inhabitants, are conditions that elevate the risk of infection.

At the time of the survey, there were 147 reported deaths due to COVID-19 in the city of Puerto Madryn, which indicates, based on the seropositivity results obtained, a fatality rate for this disease of 0.31%. The fatality rates reported by the WHO for this infection from different seroprevalence studies ranged from 0.00% to 1.63% [44].

Strengths of our study include the robust methodological design (participants selected at random, with respect to the proportion of ages and the population density per neighborhood with respect to that of the city), conducted in a city with medium population density in a low-income and midel income country (LMIC). Our results could be extrapolated to the rate of viral dissemination in cities with similar characteristics.

A large number of adults and children under 15 years of age were evaluated with a very high sensitivity and specificity test (COVIDAR IgG serological test). Our results are among the few published studies [45–48]. exploring seroprevalence for SARS Cov2 in children under 15 years of age.

One limitation of our study is that women were overrepresented (63.5 vs 36,5%) One possible explanation is that the study was conducted during the morning hours, and it is likely than men were at work at the time of the invitation to participate. Another limitation of this study was not to include in the data base the age of the children who cohabited in the same house. The risk of infection increases with the number of people living in a house, and living with children could be a risk for the spread of COVID-19.

Given the appearance of new viral variants with different levels of contagion and mortality, the data detailed here could become obsolete with the evolution of the pandemic [49–51].

This study, which shows the real difference in affected people between the SNVS 2.0 and those obtained by population serology, could be reproduced in other cities with similar characteristics: this could facilitate the identification of populations to be protected by public health interventions.

Likewise, as we see in our results, the population under 15 years of age has such a strong impact, not revealed in other studies, that it is important to deepen its evaluation in studies directed especially towards this age group.

## Conclusions

We conducted a serological survey in a medium-sized city in a LMIC, showing that the prevalence of COVID-19 infection in the population is threefold larger than the reported by the SNVS 2.0 Our results highlight the prevalence of seropositivity in children under 15 years of age and indicates that for each child infected with COVID-19, there are more than thirty unregistered infections. These data are consistent with the larger prevalence observed in various viral respiratory diseases in children as previously reported and mentioned in the discussion, including the H1N1 pandemic, but which had not been observed in the COVID-19 disease.

Our results emphasize the need for serological studies, particularly in children under 15 years of age, to objectively evaluate the real extent and spread of the SARS-CoV-2 pandemic.

## Supporting information

S1 Table Prevalence of symptoms in a subset of underage participants. (TIF)

S1 Data (XLSX)

S2 Data (XLSX)
