# Supplementary figures and images for: SARS-CoV-2 seroprevalence in the city of Puerto Madryn: Underdiagnosis and relevance of children in the pandemic
Source: PLoS One. 2022 Mar 14;17(3):e0263679. doi: 10.1371/journal.pone.0263679 (PMC8920177; doi:10.1371/journal.pone.0263679)

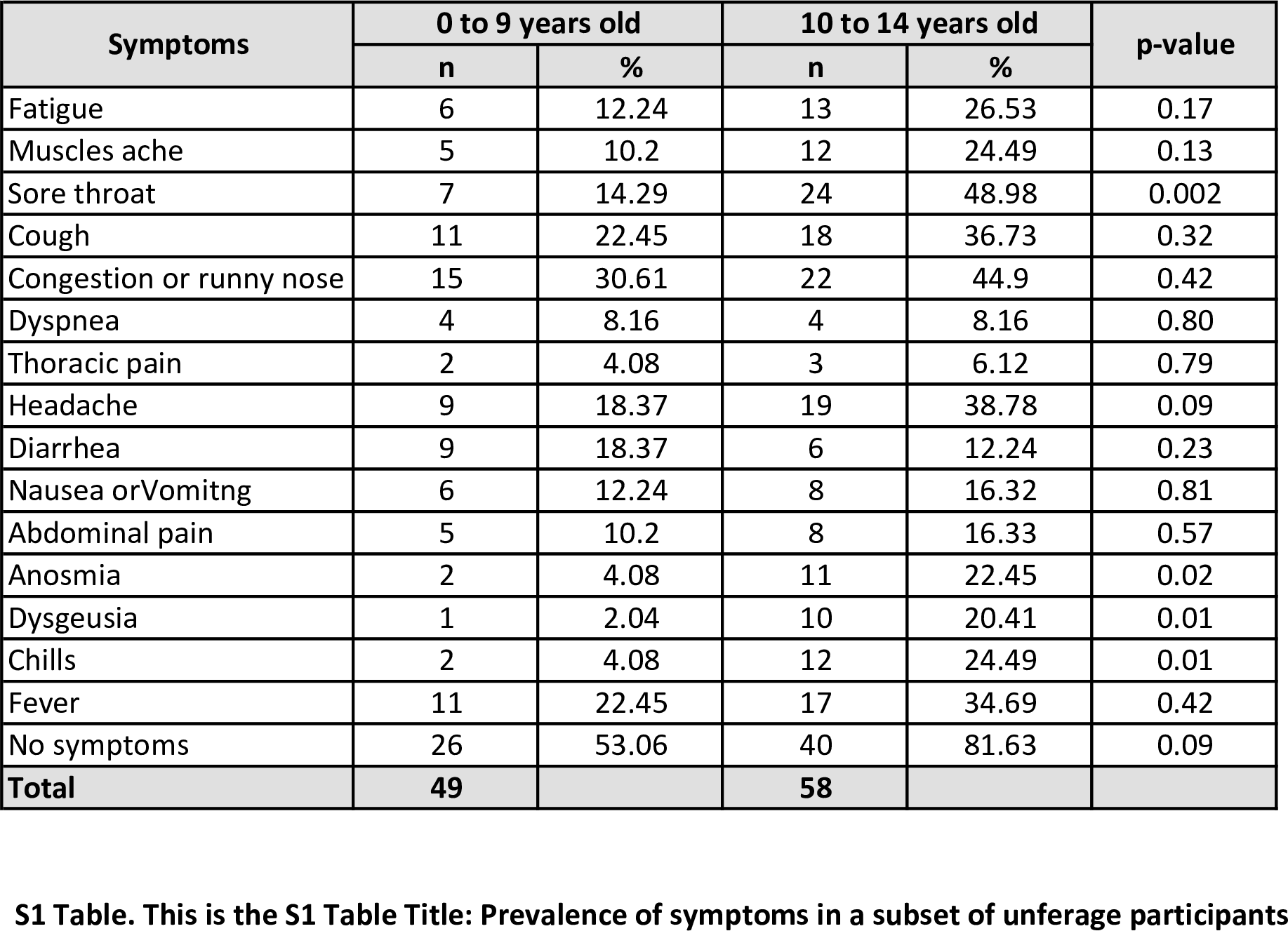

Supplement: S1 Table — (TIF) [file pone.0263679.s001.tif]
